# Correction: HMGA2, the architectural transcription factor high mobility group, is expressed in the developing and mature mouse cochlea

**DOI:** 10.1371/journal.pone.0240732

**Published:** 2020-10-13

**Authors:** Ibtihel Smeti, Isabelle Watabe, Etienne Savary, Arnaud Fontbonne, Azel Zine

## Notice of republication

This article [1] was republished on September 30, 2020, to remove an image which was not offered under a CC-BY license.

Similarities were noted between images presented in this article [1] and two additional articles published by the same research group [2, 3]:

- The schematics of postanal day-3 and adult cochlear sensory epithelia presented in Fig 1A [1] are identical to the schematics of postanal day-3 and adult cochlear sensory epithelia presented in Fig 1 [2] and the schematics presented in Gene Expression Patterns [3].
- The immunohistochemistry images of undifferentiated CGR8 mouse embryonic stem cells in Fig 1B [1] are identical to the immunohistochemistry images of undifferentiated CGR8 mouse embryonic stem cells in Fig 4D–4F [2].

The authors indicated that the schematics presented in the *PLOS ONE* articles [1, 2] are adapted from the schematic published in Gene Expression Patterns [3], which is not offered under a CC-BY license. As the original Fig 1A is not licensed for reproduction and distribution under the terms of the Creative Commons Attribution License (or Public Domain License for US gov), this content has been removed from the republished article and replaced with an alternative schematic image. Please download this article again to view the correct version.

In addition to the updated figure, additional items were clarified in the republished article:

- The figure legend of Fig 1 has been adapted so that it more accurately describes the new schematic presented in the updated version of Fig 1.
- The immunohistochemistry images presented in Fig 1B [1] and Fig 4D–4F [2] represent the same positive control for HMAG2 immunostaining. The authors indicated that the positive controls presented in these figures were not conducted simultaneously with the immunohistochemistry experiments reported in the articles but have been included for reference only (i.e. positive control) which has been clarified in the updated figure legend.
- A discrepancy was noted in the reporting of the scale bar for the immunohistochemistry images presented in Fig 1B [1] and Fig 4D–4F [2]. The figure legend for the positive control for HMAG2 immunostaining in Fig 1B [1] reports that the scale bar represents 20 μm, whereas the figure legend for the same positive control in Fig 4D–4F [2] reports a scale bar representing 50 μm. The authors have clarified that the correct measurement of the scale bar is 50 μm and the figure legend has been updated accordingly. The authors emphasize that the image has been included for reference only and that the measure of the scale bar is not essential for the overall results presented in this article.

- The authors have provided further details regarding the methodology of the embryotic stem cell experiments. The following information has been added to the Materials and Methods section: “**Culture of mouse embryonic stem cells (mESCs)** The undifferentiated mESCs (CGR8 line kindly provided by Bernard Binetruy, Aix-Marseille University, France) were expanded in the absence of feeder cells in DMEM culture medium (Gibco by Life Technologies) supplemented with LIF (leukemia inhibitory factor) on gelatin-coated plates. When the propagated cells were confluent at 80–90% (about 5–7 days), they were passaged using 0.25% trypsin-EDTA (Gibco by Life Technologies). The undifferentiated and untreated cells used for immunohistochemistry were harvested from passage 2 cell cultures and fixed in paraformaldehyde 4% in Phosphate Buffer Solution for 20 min at room temperature. After several rinses they were processed for immunohistochemistry with HMGA2 antiserum following the same immunostaining protocol used for cochlear tissue sections [21].”
- The authors have indicated that all available data are within the paper and its Supporting Information files and that the individual level data for Fig 7 are still available upon request.

## Supporting information

S1 File Originally published, uncorrected article with copyrighted image removed. (PDF)
