# Supplementary material for: Correction: HMGA2, the architectural transcription factor high mobility group, is expressed in the developing and mature mouse cochlea
Source: PLoS One. 2020 Oct 13;15(10):e0240732. doi: 10.1371/journal.pone.0240732 (PMC7553292; doi:10.1371/journal.pone.0240732)
Supplement: S1 File — (PDF) [file pone.0240732.s001.pdf]

PLOS ONE

HMGA2, the Architectural Transcription Factor High Mobility Group, Is Expressed in the Developing and Mature Mouse Cochlea

Ibtihel Smeti[1,2¤], Isabelle Watabe[1], Etienne Savary[1], Arnaud Fontbonne[1], Azel Zine[1,2]*

1 Integrative and Adaptative Neurosciences, CNRS UMR 7260 AMU, Marseille, France, **2** Sensory Biophysics, Faculty of Pharmacy, Montpellier I University, Montpellier, France

Abstract

Hmga2 protein belongs to the non-histone chromosomal high-mobility group (HMG) protein family. HMG proteins have been shown to function as architectural transcription regulators, facilitating enhanceosome formation on a variety of mammalian promoters. Hmga2 are expressed at high levels in embryonic and transformed cells. Terminally differentiated cells, however, have been reported to express only minimal, if any, Hmga2. Our previous affymetrix array data showed that Hmga2 is expressed in the developing and adult mammalian cochleas. However, the spatio-temporal expression pattern of Hmga2 in the murine cochlea remained unknown. In this study, we report the expression of Hmga2 in developing and adult cochleas using immunohistochemistry and quantitative real time PCR analysis. Immunolabeling of Hmga2 in the embryonic, postnatal, and mature cochleas showed broad Hmga2 expression in embryonic cochlea (E14.5) at the level of the developing organ of Corti in differentiating hair cells, supporting cells, in addition to immature cells in the GER and LER areas. By postnatal stage (P0–P3), Hmga2 is predominantly expressed in the hair and supporting cells, in addition to cells in the LER area. By P12, Hmga2 immunolabeling is confined to the hair cells and supporting cells. In the adult ear, Hmga2 expression is maintained in the hair and supporting cell subtypes (i.e. Deiters' cells, Hensen cells, pillar cells, inner phalangeal and border cells) in the cochlear epithelium. Using quantitative real time PCR, we found a decrease in transcript level for Hmga2 comparable to other known inner ear developmental genes (Sox2, Atoh1, Jagged1 and Hes5) in the cochlear epithelium of the adult relative to postnatal ears. These data provide for the first time the tissue-specific expression and transcription level of Hmga2 during inner ear development and suggest its potential dual role in early differentiation and maintenance of both hair and supporting cell phenotypes.

Citation: Smeti I, Watabe I, Savary E, Fontbonne A, Zine A (2014) HMGA2, the Architectural Transcription Factor High Mobility Group, Is Expressed in the Developing and Mature Mouse Cochlea. PLoS ONE 9(2): e88757. doi:10.1371/journal.pone.0088757

Editor: Berta Alsina, Universitat Pompeu Fabra, Spain

Received September 10, 2013; **Accepted** January 13, 2014; **Published** February 14, 2014

Copyright: © 2014 Smeti et al. This is an open-access article distributed under the terms of the Creative Commons Attribution License, which permits unrestricted use, distribution, and reproduction in any medium, provided the original author and source are credited.

Funding: This study was funded by ANR (Agence Nationale de Recherche; ANR-2010-BLAN-1107-01-b). The funders had no role in study design, data collection and analysis, decision to publish, or preparation of the manuscript.

Competing Interests: The authors have declared that no competing interests exist.

* E-mail: azel.zine@univ-amu.fr

¤ Current address: Laboratoire de Microorganismes et de Biomolecules, Centre de Biotechnologies, Sfax, Tunisia

Introduction

The mammalian inner ear is an intricate organ responsible for the perception of sound and balance. The first developmental process involving the mouse inner ear is the thickening of the ectoderm, known as the otic placode, next to the hindbrain at embryonic day 8.5 (E8.5) As development continues, the placode invaginates and pinches off from the surface ectoderm to form the otic vesicle at E9.5 [1,2]. Subsequently, neuroblasts delaminate from the ventral thickening of the otic vesicle and form the otic ganglion that undergoes a series of morphological changes until it reaches its mature shape by E17 [3,4]. The mammalian inner ear consists of six sensory organs: the three cristae in the semi-circular canals and the maculae in the utricle and saccule are responsible for vestibular function; the organ of Corti is responsible for auditory function. The sensory epithelia in these organs consist of sensory hair cells and non-sensory supporting cells.

The development of sensory patches in the mammalian inner ear requires complex processes of both prosensory cell specification of placode otic progenitor cells and cell fate determination [5,6]. Several lines of evidences reported that early expressed inner ear genes with long-term lasting expressions and specific effects on all or subsets of placode progenitor cells are Eya1/Six1, Pax2/8, Gata3 and Sox2 [7–9] which may help regulate neurosensory development through gene expression regulation [10,11]. While these genes have a dose and/or time dependent preferential effect on cochlear neurosensory development, Sox2 has a more dominant effect on all neurosensory precursors [12,13]. Although incomplete, a loss as in a hypomorph of Sox2 can interfere with the mammalian inner ear neurosensory precursor formation. Furthermore, it has been reported that Sox2 has a refined interaction with downstream genes such as the bHLH gene Atoh1 and shown to be required for its expression but will also be downregulated following the expression of Atoh1 in the inner ear sensory hair cells [14].

Beyond transcription factors, chromatin-remodeling regulation to allow proper transcription has been identified as a major step in neuronal specification [15] and likely plays a role in ear development as well. Among the many chromosomal transcription

Table 1. Primers pairs used for Real time PCR.

Gene name	Forward 5′→3′	Reverse 5′→3′
**Hmga2**	GTGCCACAGAAGCGAGGAC	TCTGCTTTCTTCTGGGCTGC
**Jag1**	GCTCACTTATTGCTGCGGTTG	CTTCCGCCGCTTCCTTACAC
**Hes5**	CTAATCGCCTCCAGAGCTCC	TGTCGGCCTTCTCCAGCTTG
**Sox2**	TCCAAAAACTAATCACAACAATCG	GAAGTGCAATTGGGATGAAAA
**Atoh1**	GATGAGGCCAGTTAGGAAGG	GGTAGGAGGAAGGGGATTGG
**GAPDH**	TGTCCGTCGTGGATCTGAC	CCTGCTTCACCACCTTCTTG

doi:10.1371/journal.pone.0088757.t001

regulators is the high mobility group family member Hmga2 [16–18]. This protein contains structural DNA-binding domains and act as a transcriptional regulating factor. In addition, the Hmga2 has been shown to promote maintenance of stem cell populations and proliferation by multiple means, including maintaining the expression of pluripotency genes like Sox2 and UTF [19–20].

Indeed, our previous microarray analysis of gene expression of the developing and adult cochleas identified Hmga2 and Sox2 among the differentially expressed genes between the early postnatal day-3 (P3) and adult cochlear sensory epithelia [21].

To facilitate a deeper understanding of its role in inner ear development, we present here the first comprehensive description of the expression profile of Hmga2 in the developing and adult inner ear in relation to the expression of Sox2.

These data suggest a dual role of early and long lasting Hmga2 expression in neurosensory formation and maintenance of hair and supporting cell fates.

Methods

Ethics Statement

All animal work was conducted according to the Guide to the Care and Use of Laboratory Animals [22]. Animal housing and experiments were conducted in accordance with.

French national legislation (JO 87-848) and approved by our local ethics committee named "Direction Départementale de la Protection des Populations, Préfecture des Bouches du Rhône" (France), with permit number: B13-055-25.

Animals

Wild-type mice from Swiss Webster background were used in these experiments. Female mice in mating cages were checked daily for the presence of a plug, which indicated embryonic day 1.

Fluorescent Immunohistochemistry

For embryos (E13–E14.5), whole embryos were dissected, fixed in 4% paraformaldehyde at 4°C overnight. Heads were then dissected and processed as described below.

For early neonatal (P0–P3) tissue, the animals were decapitated and their inner ears were immersed in 4% paraformaldehyde at 4°C overnight.

For postnatal (P12) and adult (>P60) tissues, the animals were killed using CO_2, and intralabyranthine perfusion of fixative 4% paraformaldehyde through the round window was quickly performed prior to immersion of the temporal bone in fixative at 4°C overnight. The inner ears were then immersed in a solution containing 10% EDTA and 1% paraformaldehyde at 4°C for 2–4 days for decalcification.

Next, the tissue was washed in phosphate-buffered saline (PBS), and incubated on ice in 10%, sucrose solution for 0.5 h or until the tissue sank, and then incubated in 20% sucrose at 4°C overnight. The tissue was then incubated in 1:1 Cryo-OCT Compound (VWR, France) and 20% sucrose at 4°C overnight. Then, the tissue was washed in 100% OCT for 10 min and then frozen, cryosectioned in 10 μm sections, and subsequently stored at −20°C.

For co-labeling experiments, the tissue cryosections were incubated in PBS supplemented with 4% normal BSA plus 0.3% Triton X-100 (Sigma) for 2 h. Next the cryosections were incubated in a solution containing 1:200 dilution of rabbit antiserum anti-Hmga2 (Dr. Narita, Cambridge Research Institute, UK) and in either 1:200 anti-Sox2 antibody (made in goat; Santa Cruz Biotechnology), 1:200 anti-MyosinVIIa antibody (made in mouse; Developmental Studies Hybridoma Bank; University of Iowa), 1:200 anti-parvalbumin antibody (made in guinea pig; Synaptic Systems) diluted in PBS supplemented with 4% BSA plus 0.1% Triton X-100 overnight at 4°C. Then, the sections were washed, and then incubated in either anti-rabbit AlexaFluor 350 or anti-rabbit AlexaFluor-568 or anti-mouse AlexaFluor-568 or anti-guinea pig AlexaFluor-568 or anti-goat AlexaFluor-488 (Molecular Probes) diluted 1:500 in PBS supplemented with 1% BSA plus 0.1% Triton X-100 for 2 h at RT.

In some experiments, the tissue cryosections were counter-stained with a phalloidin conjugated to either AlexaFluor-350 or Alexa Fluor-568 (Molecular Probes) for 30 min to reveal the cellular borders of hair and supporting cells. In a subset of sections, nuclei were counterstained with DAPI. The sections were washed, then mounted using fluorescence mounting medium (Dako). Images were recorded using either DMRB fluorescence micro-scope (Leica) or confocal microscoy LSM 710 NLO Zeiss (Carl Zeiss).

Control experiments included negative control cochlear sections (without primary antibody) and positive control (with CGR8 mouse embryonic stem cells line) that were processed in parallel.

Quantitative Real Time PCR

We performed art-PCR to compare the expression level of Hmga2 transcripts between postnatal day-3 (P3) and adult cochlear sensory epithelia. We also included four inner ear developmental genes (Sox2, Atoh1, Jagged1, and Hes5) known to be expressed in the cochlea in the art-PCR measurements. Primers were created using the Roche Universal Probe library assay center (www.universalprobelibrary.com) which utilizes Advance Primer3 to calculate optimal size, melting point, and complementarities of primer sets. A list of primers used can be found in Table 1. We first prepared three independent RNA samples for each stage (P3 and adult) using Uneasy Mini Kit (Qiagen). Each RNA sample was converted to cDNA using hexamer primers and M-MLV reverse transcriptase (Invitrogen). The PCR reactions were run in triplicate for each sample using a Light Cycler instrument (Roche) in a reaction mixture containing RNA free water, QuantiTect SYBR Green PCR Master Mix (Qiagen) at 1×0.5 μM of each of the primer pair and cDNA at 1:25 final dilution. The amplification was performed under the following conditions: initial DNA polymerase activation and DNA denaturation at 95°C for 15 min, followed by 45 cycles of 95°C for 15 s, 55°C for 20 s and 72°C for 25 s. The GAPDH gene was used as endogenous control and the data are analyzed using the delta Ct (ΔCt) method [23], where Ct is the cycle threshold, and ΔCt = Ct gene of interest − Ct of GAPDH. The gene expression levels relative to control are represented as $2E^{-\Delta ct} \times 10E^{6}$ on a $\log_{10}$ scale.

IMAGE REMOVED

Figure 1. Schematic of the postnatal day-3 (P3) and adult organs of Corti (A). inner hair cells (IHC) and outer hair cells (OHCs), as well as the non-sensory supporting cell types are indicated. Deiters' cells (DCs) surround each OHC separating them from each other and their nuclei are located underneath the nuclei of the OHCs. The pillar cells (PCs) separate the IHCs from the OHCs. Hensen's cells (HeCs) are external to the OHCs as indicated. Inner phalengeal (IPC) and border cells (BC) surround each IHC. The IHC and OHC derived from the greater epithelial ridge (GER) and the lesser epithelial ridge (LER), respectively, during embryogenesis. The cytologic changes that occur during the postnatal development within the GER area contribute to the formation of the inner sulcus (IS). The interdental cells (IDCs) are located in the limbus (LM) area of the adult organ of Corti. BM: basilar membrane. Image adapted from [47]. Undifferentiated CGR8 mouse embryonic stem (ES) cells growing in feeder-free (LIF containing medium) cell culture medium used as positive control for Hmga2 expression (B). All the nuclei of the ES cells are uniformly immunolabeled with Hmga2 antiserum (shown in red). The nuclei are counterstained with DAPI (shown in blue). Scale bar: 20 μm in all panels.
doi:10.1371/journal.pone.0088757.g001

We used Mann Whitney test from GraphPad software to analyze the RT-per data. The differences among cochlear samples were considered significant when the p-value was $\leq$0.05.

Results

Development of the organ of Corti, the auditory sense organ of mammals, involves the differentiation of two types of mechano-sensory hair cells (inner and outer hair cells; IHC, OHCs) and three types of supporting cells (Deiters's cells, inner phalangeal cells around IHCs and pillar cells separating the outer from the inner compartment). Additional cell subtypes provide the transition to the non-sensory epithelium: border cells toward the inner spiral sulcus and Hensen's cells, toward the outer spiral sulcus (Fig. 1A). Previous histological studies suggest that during cochlear embryonic development, whereas the inner hair cells derive from multivillous columnar epithelial precursor cells located in the proximal region of the greater epithelial ridge (GER), the outer hair cells derive from the most distal cells in the lesser epithelial ridge (LER). Although the general cellular pattern of the mouse organ of Corti is complete at birth [24], the onset of auditory function is estimated to occur by postnatal day-12 (P12) and adult configuration by P21 in mice [25,26]. Hmga2 has been studied in detail in the mouse where it is highly expressed in pluripotent embryonic stem (ES) cells and during embryogenesis, but is absent or expressed at low levels in adult tissues [27].

In this study, we have first used the mouse embryonic stem line CGR8 known to express high level of Hmga2 as positive control. Intense and uniform Hmga2 immunolabeling is detected in all the nuclei of undifferentiated CGR8 cells maintained under proliferating culture conditions in feeder-free (leukemia inhibitory factor containing medium). This positive control with pluripotent stem cells (Fig. 1B) in addition, to negative control (data not shown) confirmed the specificity of the Hmga2 anti-serum used in our experiments.

In order to determine the spatio-temporal expression pattern of Hmga2, sections of cochlea's of embryonic day-13 (E13) and E14.5, early postnatal (P0–P3), adolescent (P12), and adult ($\geq$P60) mice were immunolabeled with Hmga2 anti-serum alone or in combination with other inner ear cell type markers (i.e., Sox2, MyosinVIIa, Parvalbumin, Phalloidin).

At E13, no Hmga2 expression was obvious in any regions of the inner ear, except a weak signal at the level of the vestibular ganglion. In contrast, at this stage of maturation, Sox2 expression

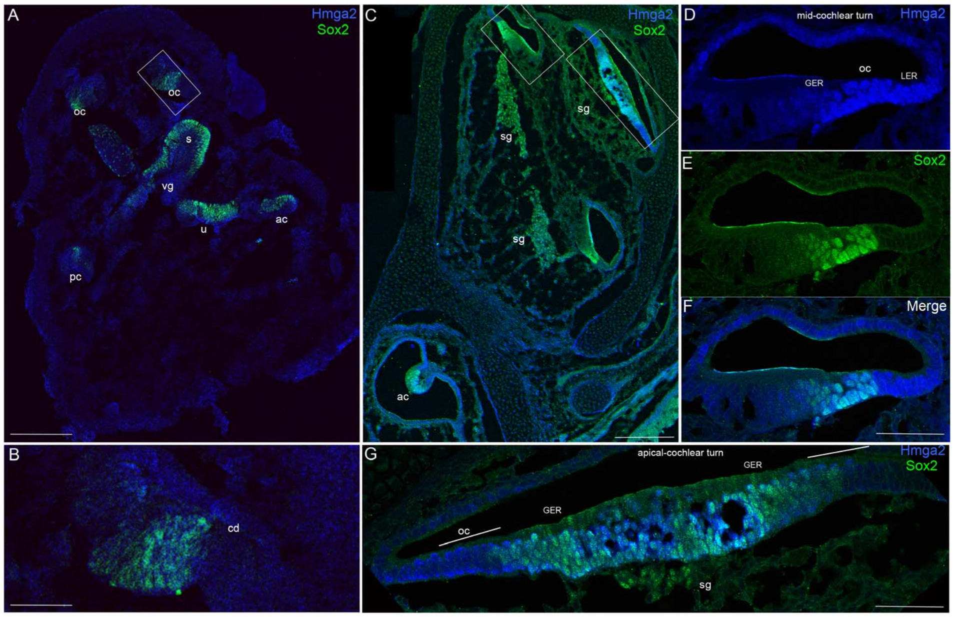

Figure 2. Hmga2 expression pattern in the embryonic E13 (A–B) and E14.5 (C–G) mouse inner ears. Low-magnification micrograph of inner ear longitudinal section immunostained with Hmga2 (shown in blue) and Sox2 (shown in green) antibodies (A). In the E13 inner ear longitudinal section, a vestibular ganglion in the boundary of the saccule have a relatively weaker Hmga2 expression, while no obvious Hmga2 signal at other regions of the inner ear. In contrast, Sox2 expression marked the prosensory domains of the cristae (ac, pc), utricular macula (u), saccular macula (s) and the organ of Corti (oc). Higher-magnification view of the inner ear longitudinal section (B) at the level of the region indicated by the box in (A). In the developing cochlear duct (cd), Sox2 marked the prosensory region of the organ of Corti. No obvious signal for Hmga2 expression was detected in this Sox2 prosensory region. Low-magnification micrograph of E14.5 inner ear longitudinal section immunostained with Hmga2 (shown in blue) and Sox2 (shown in green) antibodies (C). (D–F) Higher-magnification view of the inner ear longitudinal section at the level of the mid-cochlear turn in the region indicated by the small box in (C). Hmga2 expression is observed in the nuclei of hair cells and supporting cells of the organ of Corti's and in the GER and LER cells. In the OC, Hmga2 (D,F, blue) co-localized with Sox2 (E,F, green) in the hair cells and supporting cells. (G) Higher-magnification view of the inner ear longitudinal section through the apical cochlear turn in the region indicated by the large box in (C). Hmga2 immunolabeling is localized to lateral halves of the cochlear duct (the site of the future OC) and was present in the GER throughout the thickness of the epithelium (shown in Blue) where it is colocalized with Sox2 (shown in green). Organ of Corti (OC), spiral ganglion (sg); vestibular ganglion (vg); cochlear duct (cd); lesser epithelial ridge (LER); greater epithelial ridge (GER), anterior crista (ac); posterior crista (pc). Scale bars = 200 µm in (A,C) and 50 µm in (B,D,E,F,G).
doi:10.1371/journal.pone.0088757.g002

marked the sensory primordia of the cristae, maculae and cochlear duct (Fig. 2A–B). Double labeling with Sox2 (Fig. 2–3) which labels in the embryo the developing prosensory domain of the otocyst and statoacoustic ganglion [12,13] confirmed that Hmga2 is expressed in this tissue.

We were first able to detect Hmga2 immunoreactivity in the mouse cochlear epithelium at E14.5 (Fig. 2C–G; 3A–C). Hmga2 immnolabeling was nuclear and spanned the depth of the sensory epithelium. The Hmga2 immunoreactive cells constitute a subset of the cells within the Sox2 prosensory domain and immature cells in the GER and LER regions (Fig. 2F–G). At this time point, Hmga2 signal is slightly higher in the vestibular epithelium (i.e., anterior crista, Fig. 3C) than the cochlear epithelium.

At E14.5, Hmga2 immunolabeling was also present in the nuclei of both the nascent inner hair cells and supporting cells of the developing cochlear and vestibular systems. In the cochlea of the embryo, Hmga2 was expressed in a gradient along the length of the cochlea. In the apical turns (Fig. 2G, 3A), Hmga2 expression is slightly lower than the middle (Fig. 2F, 3B) and most basal turns (data not shown).

We also detected Hmga2 expression in both the developing spiral and vestibular ganglia (Fig. 3B–C); however, this expression is much weaker than that seen in the developing sensory epithelia at the same stage of maturation. Thus by E14.5, Hmga2 immunolabeling was present in the developing cochlea, vestibular organs and statoacoustic ganglion.

After birth (P0–P3), Hmga2 immunolabeling was downregulated in the spiral ganglion, vestibular sensory epithelia (data not shown), and in the GER immature cells located medially to the organ of Corti.

Between P0 and P3, the different supporting cells are easily distinguished by their position relative to the inner and outer hair cells and the basilar membrane (Fig. 4–5).

During this early postnatal stage, strong Hmga2 immunolabeling is detected in the inner and outer hair cells, in addition to Deiters', inner phalengeal, pillar cell nuclei, in addition to the LER area (Fig. 4C, 5B). In contrast to the downregulation in GER cells, Hmga2 continues to be expressed in immature cells of the LER region located laterally to the organ of Corti.

In the sensory epithelium of early postnatal cochlea, Hmga2 was expressed in a gradient fashion along the length of the cochlea. In the apical turn (Fig. 4C), the hair and supporting cells exhibited a slightly stronger Hmga2 immunolabeling as compared to the basal turn (Fig. 5B). In contrast to the embryo expression pattern, in the postnatal stage, Sox2 is downregulated from the hair cells and the co-localization with Hmga2 was limited to the inner ear supporting cells (Fig.4–5).

By P12, which is the approximate period of the onset of organ of Corti's function in mice [25,28], Hmga2 immunolabeling was observed in both inner hair cells and outer hair cells and different supporting cell subtypes (Deiter's cells, Hensen cells, pillar cells, inner phalangeal cells and border cells) (Fig. 6). Double labeling

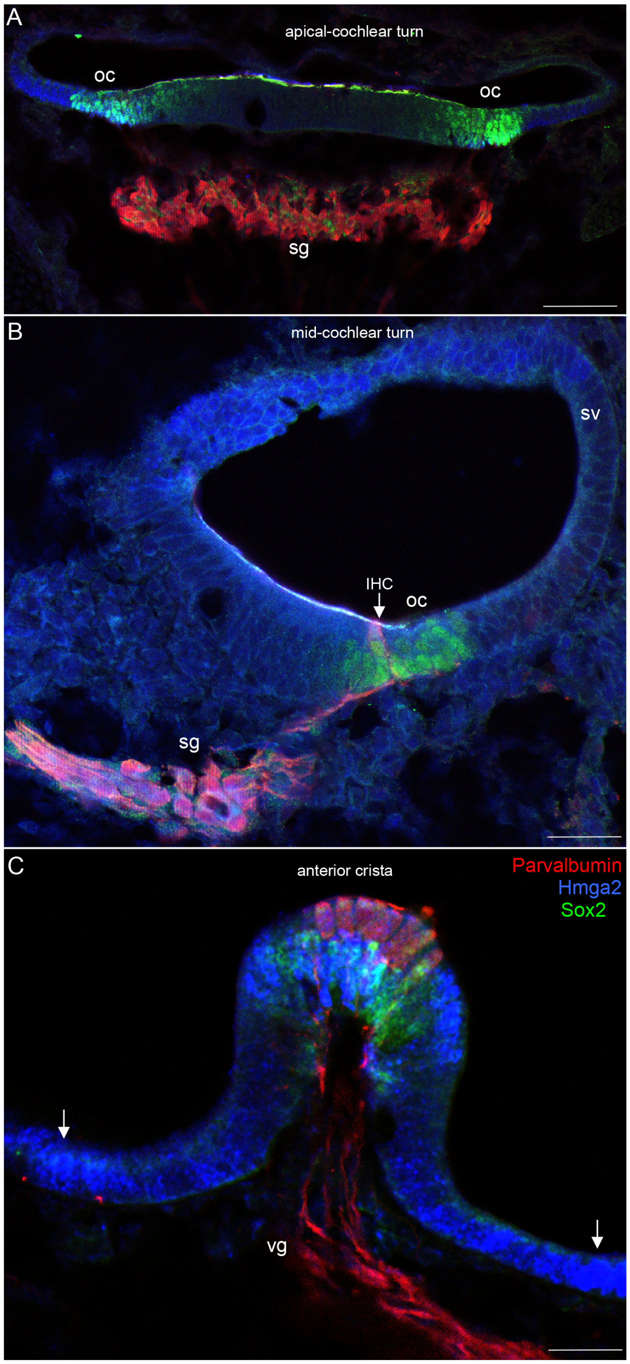

Figure 3. At E14.5, Hmga2 is expressed in the developing inner ear sensory epithelia of the cochlea (A–B) and the vestibule (C). In the cochlea, Hmga2 (shown in blue) is co-expressed with Sox2 (shown in green) expressing-cells in the organ of Corti. Hmga2 expression colocalized with Sox2 in the prosensory domain and no sign of hair cell differentiation at the apical cochlear turn at this stage of maturation (A). The spiral ganglion is immunostained by Parvalbumin while Hmga2 signal is not obvious. In the mid-cochlear turn (B), Parvalbumin (shown in red) labeled the differentiating inner hair cell (IHC) and the spiral ganglion where it is colocalized with Sox2. In the developing vestibule, Hmga2 (C, Blue) immunolabeling partially overlapped with Parvalbumin (C, red) in the hair cells at the lumenal layer and with Sox2 (C, green) in the supporting cells at the basal layer of the anterior crista. The cells located in the extrastriolar regions showed a strong Hmga2 signal (arrows). Parvalbumin labels both hair cells and ganglion neurons in the cochlea and vestibule. spiral ganglion (sg); vestibular ganglion (vg); stria vascularis (sv). Scale bars = 100 μm in all panels.
doi:10.1371/journal.pone.0088757.g003

with either Myosin VIIa (hair cell marker) or fluorescent-phalloidin (marker of stereocilia and cellular borders of the organ of Corti) and Sox2 at this postnatal age confirmed that Hmga2 immunolabeled both the hair cells and supporting cells in the organ of Corti's (Fig. 6A–C). In the adult cochlea (i.e., P60), Hmga2 immunolabeling intensity although slightly decreased was maintained in the nuclei of both hair cells and in different supporting cell subtypes in the organ of Corti (Fig. 6D).

To confirm the immunohistochemistry findings, Hmga2 expression in the cochlear sensory epithelium was measured using quantitative real time PCR (qRT-PCR) at two key developmental stages (i.e., P3 and P60) of the mouse inner ear. The qRT-PCR results (Fig. 7) indicated that similar to the expression levels of other known inner ear differentiation genes (i.e. Sox2, Atoh1, Jagged1 and Hes5), Hmga2 transcripts are downregulated in the cochlear sensory epithelium of the adult (P60) when compared to the early postnatal (P3) mice.

Discussion

These results indicate that Hmga2 is expressed in the developing cochlea and that its expression level changes as the cochlea matures. We show that Hmga2 is broadly expressed in both the developing cochlea, vestibule and statoacoustic ganglion in the murine embryo. In addition, postnatal and adult inner ear sensory hair cells and their non-sensory supporting cells, which are terminally differentiated cells, express Hmga2; however, its transcriptional level decreases in the adult cochlear sensory epithelium as revealed by qRT-PCR measurements.

Different populations of non-sensory supporting cells in the early postnatal, adolescent, and adult cochleas exhibited Hmga2 immunolabeling in the pillar cells and in the supporting cells in direct contact with inner hair cells (border cells and inner phalangeal cells) and outer hair cells (Deiters's and Hensen cells).

The sensory cochlear hair cells expressed Hmga2 from the onset of hair cell differentiation in the mouse embryo by E14.5 [29,30] through the adult (P60). This is in contrast with the expression of Sox2 which is also expressed in both developing hair cells and supporting cells until early postnatal day-2 (P2), but its expression is maintained only in the supporting cells of postnatal and adult mice [12–14]. However, Hmga2 and Sox2 expression overlapped in both the hair cells and supporting cells in the embryo and overlapped only in the supporting cells in postnatal and adult mice. Furthermore, the intensity of Hmga2 labeling exhibits a base-to-apex gradient, with a relatively stronger immunolabeling in the hair and supporting cells in the apical turns compared with the basal turn of the early postnatal cochleas.

Finally, Hmga2 labels also both hair cells and supporting cells in the embryonic and adult vestibular system of the murine inner ear.

It has been previously demonstrated that Hmga2 is highly expressed in undifferentiated cells during embryogenesis, in neural stem cells, in transformed cells and barely detectable in normal adult tissue [16,31,32].

Given that Hmga2 acts as architectural transcription factor linked to proliferation and expressed in pluripotent embryonic stem cells and neural stem cells [31–33], it is surprisingly to detect its expression in the postnatal and adult mouse inner ear sensory hair cells and their supporting cells, which are terminally differentiated cells at this maturation stage.

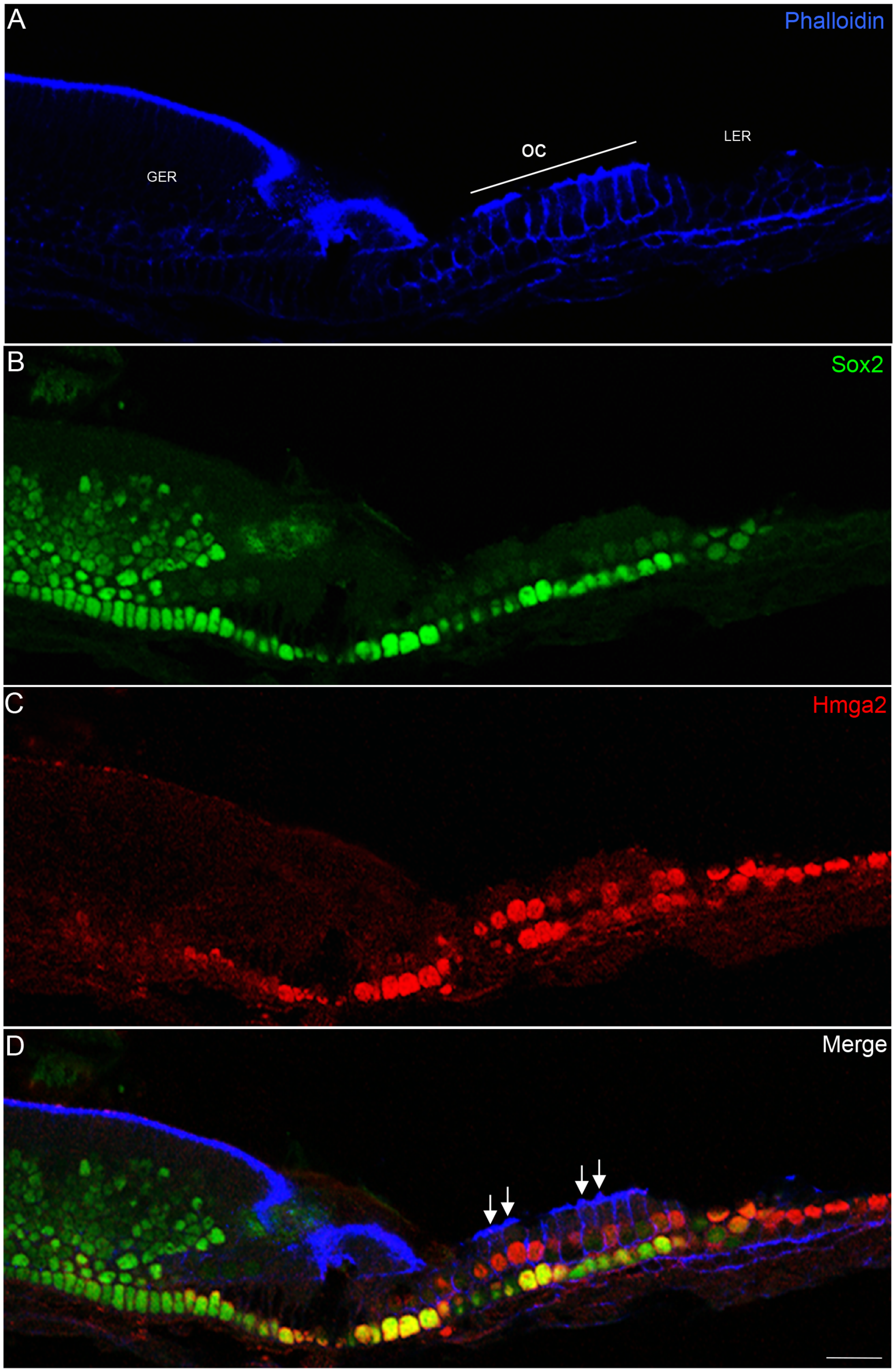

Figure 4. Hmga2 immunostaining in the newborn cochlea. Tangential section through the mid-turn (z-section) of the cochlea showing widespread expression of Hmga2 (shown in red) in the nuclei of hair cells and supporting cells; in the LER region; Deiter's cells and in pillar cells. In the organ of Corti area, Hmga2 is colocalized (shown in yellow) with Sox2 in the supporting cells. Phalloidin marker (shown in blue) was used to outline the general structure of the cochlear epithelium including stereocilia (D, arrows) and the cellular borders of hair and supporting cells. Scale bars = 20 µm in all panels.
doi:10.1371/journal.pone.0088757.g004

The Sox2 is another stem cell marker linked to proliferation and differentiation within the inner ear sensory epithelia and similar to Hmga2, its expression spanned a whole development including both the embryonic and mature inner ear stages [34,35]. Interestingly, it has been suggested that Hmga2 plays a dose-dependent role in the inversely correlated phenomena of stemness and differentiation [18] as what was reported for the dose-dependent function of Sox2 in different cell lineages [36,37].

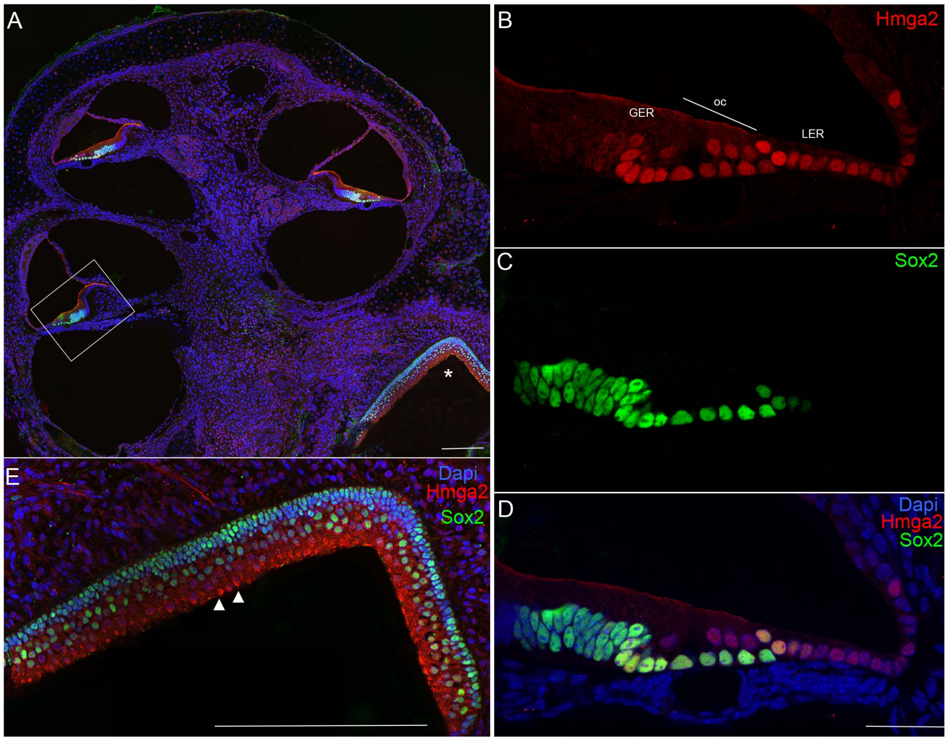

Figure 5. Hmga2 expression in postnatal day-3 (P3) inner ear (A–D). Low-magnification micrograph of inner ear longitudinal section (A) immunostained with Hmga2 (shown in red) and Sox2 (shown in green). Higher-magnification view at the level of the basal cochlear turn (B–D) in the region indicated by the box in (A). Hmga2 expression is observed in the nuclei of hair cells and different supporting cell subtypes. Hmga2 is colocalized with Sox2 in the supporting cells within the organ of Corti's area (shown in yellow). Intense Sox2 signal (shown in green) is also observed in cells of the GER. Higher-magnification of the utricular macula (E) in the region indicated by asterix in (A) showing moderate Hmga2 (shown in red) expression at the lumenal hair cell layer while Sox2 (shown in green) expression is restricted to supporting cells at basal layer of the macula. In all panels, cell nuclei are counterstained with DAPI (blue). Scale bars = 100 µm in (A,E) and 50 µm in (B–D).
doi:10.1371/journal.pone.0088757.g005

Although, Hmga2 is downregulated in the adult cochlea, its extended and overlapped expression with Sox2 may suggest a possible dual role of Hmga2 during inner ear development. Hmga2 may be involved first, in establishing otic progenitor cells in the prosensory domain in the embryo and then, in subsequent

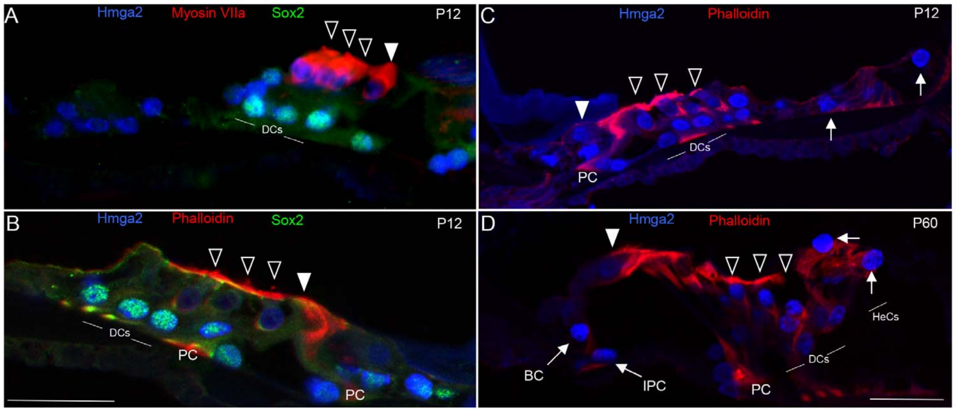

Figure 6. Hmga2 expression is maintained with Sox2 in postnatal day-12 (P12) and in adult (P60) cochleas. By P12 (A–C), Hmga2 expression (shown in blue) is still observed in the nuclei of both hair cells co-labeled with the Myosin VIIa (shown in red, A) and supporting cells co-labeled with Sox2 (shown in green, A–B). A transverse section of P12 organ of Corti's in mid-cochlear turn (C) co-labeled with Hmga2 (shown in blue) and phalloidin (shown in red) confirming the expression of Hmga2 in the hair and supporting cells. In the adult (D), Hmga2 immunolabeling (shown in blue) is maintained in the nuclei of hair and supporting cells of the organ of Corti. The supporting cells immunolabeled with Hmga2 included the Deiters cells (DCs), Pillar cells (PCs), inner phalengeal cells (IPC), Border cells (BC) and Hensen cells (HeCs, arrows). The organ of Corti outline is visualized by phalloidin-labeling (red color, B–D). Open arrowheads indicate the OHCs and filled arrowhead indicates the IHC (A–D). Scale bars = 20 µm in (A) all panels.
doi:10.1371/journal.pone.0088757.g006

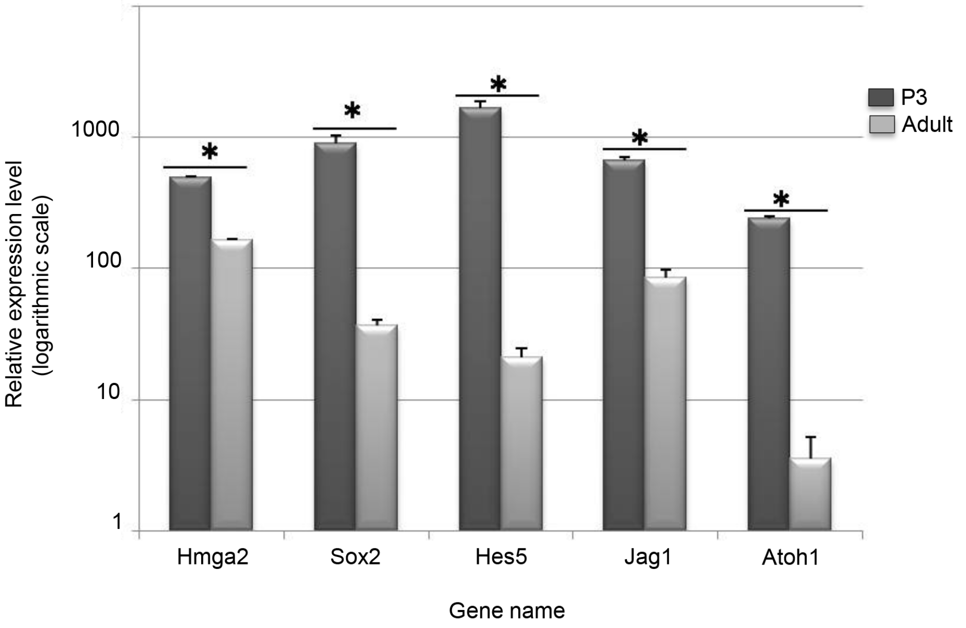

Figure 7. Down-regulation of Hmga2 and some known developmental inner ear genes (Sox2, Atoh1, Jag1 and Hes5) in the adult cochlea as analyzed by quantitative real time PCR. All of RT-q-PCR results were normalized to the expression of a reference gene GAPDH when comparing the cochlear sensory epithelia from adult and P3 mice. Three independent triplicate biological samples from each stage (Adult vs. P3) were used for the q-PCR measurements. A Mann Whitney test was performed on q-PCR results. * Indicates significant p value ≤ 0.05.
doi:10.1371/journal.pone.0088757.g007

differentiation and maintenance of hair cells and supporting cells in postnatal and adult cochleas.

Furthermore, it has been shown that Hmga2 promote the maintenance of different stem cell populations and proliferation by multiple means, including maintaining the expression of pluripotency genes like Sox2, UTF1 and Oct4 [18,38], enhancing E2F1 activity [20]. Indeed, Hmga2 promotes neural stem cell self-renewal by reducing expression of two negative regulators of the cell cycle, p16Ink4a and p19Arf [31].

Moving upstream from p16INK4a and Hmga2 signaling pathways, one may find that both microRNA and histone acetylation have a demonstrated role in Hmga2 expression levels.

Indeed, during oncogenic transformation process, microRNA levels of the translation-regulatory factor let-7 inversely correlate with expression of the Hmga2. In a mouse model, several reports demonstrate that members of the let7 family of miRs target Hmga2 resulting in its posttranscriptional downregulation [39–41]. Thus let7 miRs are potential targets for preventing proliferation not only via the loss of Hmga2 and all of its downstream effects, but also via the degradation of the known oncogene hRas [41].

Interestingly, the expression of several let7 miRs are significantly decreased during hair cell regeneration in the adult newt [42], suggesting that let-7 miRNAs may indeed be important for the maintenance of quiescence in inner ear mechanosensory epithelia. Furthermore, it has been shown that several let-7 family members are expressed in the postnatal murine cochlea, and that their expression levels persist up to at least P100 [43], suggesting that let7miRs could negatively regulate Hmga2 in the postnatal and adult cochleas and prevent both hair cells and supporting cells proliferation.

The histone deacetylase enzymes (HDACs) have been also shown to play a role in the regulation of Hmga2, where inhibition of HDACs with the HDAC inhibitor Trichostatin A results in transcriptional repression of the Hmga2 gene in several different cell lines [44]. This is may be important as the *in vitro* application of HDAC inhibitors to regenerating chicken utricles significantly reduced the numbers of proliferating supporting cells [45].

In addition, another chromatin remodeling factor, the SWI/SNF complex has been reported to be involved in the transcriptional activation that regulates neuronal development in the mammalian inner ear in cooperation with Eya/Six1 and Sox2 [46].

Finally, this is the first quantitative real time PCR data comparing the adult with the early postnatal inner ear. Our data suggest a decrease, but not a loss as suggested by some previous immunocytochemical data, of embryonic transcription factors such as Sox2 and Atoh1. These data suggest that the embryonic transcription regulation system remains active but at a much reduced level in the mouse adult inner ear.

The demonstrated effects of Hmga2 on numerous factors known to affect cell proliferation and stem cell self-renewal, suggest Hmga2 as an attractive potential target gene for manipulation in the mouse cochlea through loss and gain-of-function studies to ascertain whether or not its transcriptional downregulation with age could be in relation with the known *in vivo* quiescence of postmitotic sensory hair cells and supporting cells in the adult cochlea. However, more work is required to identify the functional role of Hmga2 and the type of its possible interaction with Sox2 in the mammalian cochlea.

Acknowledgments

We thank Dr. B. Fritsch (University of Iowa, USA) for his critical reading, Dr. B. Binetruy (INSERM, UMR S-910, AMU, France) for the ES CGR8 cells, and Dr. M. Narita (Cambridge Institute, UK) for the HMGA2 anti-serum. We thank A. Tonetto and C. Hecquet (Microscopy facility, AMU, France) for their excellent assistance with confocal imaging. We also thank Dr. P. Ravel (Faculty of Pharmacy, University of Montpellier I, France) for help with statistical analysis.

Author Contributions

Conceived and designed the experiments: AZ. Performed the experiments: IS IW ES AF. Analyzed the data: IS AZ. Contributed reagents/materials/analysis tools: IS AZ. Wrote the paper: IS AZ.

References

1. Barald KF, Kelley M (2004) From placode to polarization: new tunes in inner ear development. Development 131: 4119 4130.
2. Chen J, Streit A (2013) Induction of the inner ear: stepwise specification of otic fate from multipotent progenitors. Hear Res 297: 3 12.
3. Morsli H, Choo D, Ryan A, Johnson R, Wu DK (1998) Development of the mouse inner ear and origin of its sensory organs. J. Neurosci 18: 3327 3335.
4. Kopecky BJ, Duncan JS, Elliott KL, Fritzsch B (2012) Three-dimensional reconstructions from optical sections of thick mouse inner ears using confocal microscopy. J Microsc 248: 292 298.
5. Fritzsch B, Beisel KW, Hansen LA (2006) The molecular basis of neurosensory cell formation in ear development: a blueprint for hair cell and sensory neuron regeneration? Bioessays 28: 1181 1193.
6. Kelley MW (2007) Cellular commitment and differentiation in the organ of Corti. Int J Dev Biol (6–7): 571 83.
7. Zou D, Erickson C, Kim EH, Jin D, Fritzsch B, et al. (2008) Eya1 gene dosage critically affects the development of sensory epithelia in the mammalian inner ear. Hum Mol Genet 17: 3340 3356.
8. Bouchard M, de Caprona D, Busslinger M, Xu P, Fritzsch B (2010) Pax2 and Pax8 cooperate in mouse inner ear morphogenesis and innervation. BMC Dev Biol 10: 89.
9. Duncan JS, Fritzsch B (2013) Continued expression of GATA3 is necessary for cochlear neurosensory development. PLoS One 8(4): e62046.
10. Ahmed M, Xu J, Xu PX (2012) EYA1-SIX1 complex in neurosensory cell fate induction in the mammalian inner ear. Development 139: 1965–77.
11. Wong EY, Ahmed M, Xu PX (2013) EYA1-SIX1 complex in neurosensory cell fate induction in the mammalian inner ear. Hear Res 297: 13 19.
12. Kiernan AE, Pelling AL, Leung KK, Tang AS, Bell DM, et al. (2005) Sox2 is required for sensory organ development in the mammalian inner ear. Nature 434: 1031 1035.
13. Puligilla C, Dabdoub A, Brenowitz SD, Kelley MW (2010) Sox2 induces neuronal formation in the developing mammalian cochlea. J Neurosci 30: 714 722.
14. Dabdoub A, Puligilla C, Jones JM, Fritzsch B, Cheah KS, et al. (2008) Sox2 signaling in prosensory domain specification and subsequent hair cell differentiation in the developing cochlea. Proc Natl Acad Sci USA 105: 18396 18401.
15. Ronan JL, Wu W, Crabtree GR (2013) From neural development to cognition: unexpected roles for chromatin. Nat Rev Genet 14: 347 359.
16. Reeves R (2001) Molecular biology of HMGA proteins: hubs of nuclear function. Gene 277: 63 81.
17. Cleynen I, Van De Ven JM (2008) The HMGA proteins: A myriad of functions. International Journal of Oncology 32: 289 305.
18. Malini E, Maurizio E, Bembich S, Sgarra R, Edomi P, et al. (2011) HMGA Interactome: new insights from phage display technology. Biochemistry 50: 3462 8.
19. Li O, Li J, Dröge P (2007) DNA architectural factor and proto-oncogene HMGA2 regulates key developmental genes in pluripotent human embryonic stem cells. FEBS Lett 581: 3533 3537.
20. Fedele M, Visone R, De Martino I, Troncone G, Palmieri D, et al. (2006) HMGA2 induces pituitary tumorigenesis by enhancing E2F1 activity. Cancer Cell 9: 459 471.
21. Smeti I, Assou S, Savary E, Masmoudi S, Zine A (2012) Transcriptomic analysis of the developing and adult mouse cochlear sensory epithelia. PloS One 7: e42987.
22. National Center for Biotechnology Information website. Guide for the Care and Use of Laboratory Animals -NCBI Bookshelf Available: http://www.ncbi.nlm.nih.gov/books/NBK54050/.
23. Livak KJ, Schmittgen TD (2001) Analysis of relative gene expression data using real- time quantitative PCR and the 2(-Delta Delta C(T)) Method. Methods 25: 402 408.
24. Lim DJ (1986) Functional structure of the organ of Corti: a review. Hear Res 22: 117 146.
25. Hinojosa (1977) A note on development of Corti's organ. Acta Otolaryngol 84: 238 251.
26. Lim DJ, Rueda J (1992) In: Structural Development of the Cochlea. Elsevier, New York, 33 58.
27. Hirning-Folz U, Wilda M, Rippe V, Bullerdiek J, Hameister H (1998) The expression pattern of the Hmgic gene during development. Genes Chromosomes Cancer 23: 350 357.
28. Pujol R, Hilding D (1973) Anatomy and physiology of the onset of auditory function. Acta Otolaryngol 76: 1 10.
29. Chen P, Johnson JE, Zoghbi HY, Segil N (2002) The role of Math1 in inner ear development: uncoupling the establishment of the sensory primordium from hair cell fate determination. Development 129: 2495 2505.
30. Kelley MW (2006) Regulation of cell fate in the sensory epithelia of the inner ear. Nat. Rev. Neurosci 7: 837 849.
31. Nishino J, Kim I, Chada K, Morrison SJ (2008) Hmga2 promotes neural stem cell self- renewal in young but not old mice by reducing p16Ink4a and p19Arf Expression. Cell 135: 227 239.
32. Fedele M, Palmieri D, Fusco A (2010) HMGA2: A pituitary tumour subtype-specific oncogene? Mol Cell Endocrinol 326: 19 2.
33. Hammond SM, Sharpless NE (2008) HMGA2, MicroRNAs, and Stem Cell Aging. Cell 135: 1013 1016.
34. Hume CR, Bratt DL, Oesterle EC (2007) Expression of LHX3 and SOX2 during mouse inner ear development. Gene Expr Patterns 7: 798 807.
35. Mak AC, Szeto IY, Fritzsch B, Cheah KS (2009) Differential and overlapping expression pattern of SOX2 and SOX9 in inner ear development. Gene Expr Patterns 9: 444 453.
36. Taranova OV, Magness ST, Fagan BM, Wu Y, Surzenko N, et al. (2006) SOX2 is a dose-dependent regulator of retinal neural progenitor competence. Genes Dev 20: 1187 1202.
37. Seo E, Basu-Roy U, Zavadil J, Basilico C, Mansukhani A (2011) Distinct functions of Sox2 control self-renewal and differentiation in the osteoblast lineage. Mol Cell Biol 22: 4593 4608.
38. Tay Y, Peter S, Rigoutsos I, Barahona P, Ahmed S, et al. (2009) Insights into the Regulation of a Common Variant of HMGA2 Associated with Human Height During Embryonic Development. Stem Cell Rev Rep 5: 328 333.
39. Mayr C, Hemann MT, Bartel DP (2007) Disrupting the pairing between let-7 and Hmga2 enhances oncogenic transformation. Science 315: 1576 1579.
40. Lee YS, Dutta A (2007) The tumor suppressor microRNA let-7 represses the HMGA2 oncogene. Genes & Development 21: 1025 1030.
41. Yu F, Yao H, Zhu P, Zhang X, Pan Q, et al. (2007) Let-7 regulates self renewal and tumorigenicity of breast cancer cells. Cell 131: 1109 1123.
42. Tsonis PA, Call MK, Grogg MW, Sartor MA, Taylor RR, et al. (2007) MicroRNAs and regeneration: let-7 members as potential regulators of dedifferentiation in lens and inner ear hair cell regeneration of the adult newt. Biochemical and Biophysical Research Communications 362: 940 945.
43. Weston MD, Pierce ML, Rocha-Sanchez S, Beisel KW, Soukup GA (2006) MicroRNA gene expression in the mouse inner ear. Brain Research 1111: 95 104.
44. Ferguson M, Henry PA, Currie RA (2003) Histone deacetylase inhibition is associated with transcriptional repression of the Hmga2 gene. Nucleic Acids Research 31: 3123 3133.
45. Slattery EL, Speck JD, Warchol ME (2009) Epigenetic influences on sensory regeneration: histone deacetylases regulate supporting cell proliferation in the avian utricle. Journal of the Association for Research in Otolaryngology 10: 341 353.
46. Ahmed M, Xu J, Xu PX (2012) EYA1 and SIX1 drive the neuronal developmental program in cooperation with the SWI/SNF chromatin-remodeling complex and SOX2 in the mammalian inner ear. Development 139: 1965 77.
47. Smeti I, Savary E, Capelle V, Hugnot JP, Uziel A, et al. (2011) Expression of candidate markers for stem/progenitor cells in the inner ears of developing andadult GFAP and nestin promoter-GFP transgenic mice. Gene Expression Patterns 11: 22 32.
